# Qualitative and quantitative CEUS assessment for differential diagnosis in thyrotoxicosis: destruction vs. hyperfunction

**DOI:** 10.1007/s12020-025-04324-6

**Published:** 2025-06-28

**Authors:** Dana Stoian, Luciana Moisa-Luca, Andreea Bena

**Affiliations:** 1 Discipline of Endocrinology, Second Department of Internal Medicine, University of Medicine and Pharmacy “Victor Babes”, Timisoara, Romania; 2 Center for Molecular Research in Nephrology and Vascular Disease, “Victor Babes” University of Medicine and Pharmacy, Timisoara, Romania

**Keywords:** Thyrotoxicosis, Quantitative CEUS, Destruction, Time-intensity curve

## Abstract

**Purpose:**

Thyrotoxicosis results from either thyroid hyperfunction, as seen in Graves’ disease, or tissue destruction, as in subacute thyroiditis (SAT). Differential diagnosis is essential but can be challenging, with thyroid scintigraphy traditionally used to distinguish between these types. This study investigates the potential of Contrast-Enhanced Ultrasound (CEUS) to provide a non-invasive alternative for differentiating hyperfunction and destructive thyroid disease.

**Methods:**

In a prospective study, 42 patients with untreated thyrotoxicosis were evaluated using CEUS to analyze both qualitative and quantitative perfusion parameters.

**Results:**

Group 1 included 20 patients with Graves’ disease, while Group 2 comprised 22 patients with destructive thyrotoxicosis (SAT, Hashitoxicosis, postpartum thyroiditis, and iatrogenic cases). Significant differences were observed in CEUS parameters between the two groups. Hyperfunction cases showed higher Peak Intensity (PKI), Area Under Curve (AUC), Wash-in AUC (WiAUC), Wash-out AUC (WoAUC), and Mean Time-Intensity Curve (mTIC), while Rise Time (RT) and Time to Peak (TTP) were prolonged in the destruction group. Strong correlations with hyperfunction were observed for PKI, AUC, WoAUC, and mTIC. Optimal cut-off values of AUC > 1991, WoAUC > 1876, and mTIC > 10 achieved 100% sensitivity and specificity for identifying hyperfunction.

**Conclusion:**

This preliminary study suggests that CEUS, with its quantitative and qualitative assessments, could be a valuable tool in the non-invasive differential diagnosis of thyrotoxicosis, potentially reducing the need for scintigraphy and enabling rapid and accurate treatment decisions.

## Introduction

Thyrotoxicosis is characterized by an increased metabolic rate, primarily driven by increased levels of free thyroxine (T4) and triiodothyronine (T3) in the bloodstream [1]. Common causes include either thyroid follicle overstimulation, hyperplastic growth of the thyroid epithelium, or sudden damage to follicular cells, often seen in inflammatory thyroid conditions or metastatic invasions. Beyond intrinsic thyroid pathology, various pharmaceuticals and cancer therapies are also implicated in disturbing thyroid homeostasis, leading to increased hormonal activity [1–4]. Diffuse thyroid disease (DTD) encompasses a range of thyroid pathologies of diverse etiology, characterized by diffuse alteration of the thyroid parenchyma structure, leading to thyroid dysfunction: hypothyroidism and thyrotoxicosis, respectively. Among these, autoimmune thyroid diseases have the highest prevalence and are represented by chronic autoimmune thyroiditis, and Graves’ disease (GD) [5]. On the other hand, impaired thyroid function may result from destructive mechanisms affecting thyroid follicles due to viral infections (subacute thyroiditis), medication use (amiodarone, lithium, interleukin-2, interferon-alpha, immunotherapy), or the postpartum period (postpartum thyroiditis) [6].

Differential diagnosis of thyrotoxicosis can be complex, as the underlying cause is not always straightforward. Thyroid receptor antibodies (TRAb) are highly indicative of Graves’ disease [7], particularly when diffuse hypoechogenicity and increased vascularity are observed on color Doppler ultrasound [8, 9]. Elevated C-reactive protein (CRP) levels, along with clinical signs like thyroid swelling, pain, and tenderness, are often associated with subacute thyroiditis, which appears as hypoechoic areas on ultrasound and stiff on elastography [10]. A patient’s medication history, recent postpartum period, or other relevant background factors may also help clarify the diagnosis. Although the fT3/fT4 ratio is often used as an indicator of hyperfunction rather than destructive processes, it can sometimes be misleading. While thyroid scintigraphy is considered the gold standard for distinguishing between these forms, its use is limited by accessibility issues, patient hesitation, and contraindications during pregnancy and often in the postpartum or lactating period [7, 11].

Ultrasound is an essential tool in evaluating thyrotoxicosis, particularly in distinguishing between various underlying causes such as autoimmune thyroid disease and subacute thyroiditis [12–14]. This article focuses on diffuse thyroid disease (DTD), and toxic goiter will not be discussed here. Ultrasound provides a non-invasive, accessible means of assessing thyroid morphology and vascularity, both of which are critical in differential diagnosis [15, 16]. Autoimmune thyroid diseases, like Graves’ disease, often present with characteristic findings, such as diffuse thyroid enlargement and increased vascularity on color Doppler, indicative of hyperfunction [9, 17]. Conversely, forms with gland destruction, such as subacute thyroiditis, may show hypoechoic areas without increased vascular flow and stiff on elastography, differentiating them from hyperfunctional states [10]. These opposing vascular dynamics reflect underlying pathophysiological mechanisms: in hyperfunctioning states like Graves’ disease, continuous TSH receptor stimulation leads to increased glandular blood flow and hyperemia, while destructive processes such as thyroiditis result in cellular breakdown, inflammation, and reduced perfusion. While Doppler ultrasound and elastography can aid in differentiating thyrotoxicosis subtypes, their diagnostic accuracy varies due to operator dependence and lack of standardization. This highlights the need for more objective tools like Contrast-Enhanced Ultrasound (CEUS), which may offer improved diagnostic precision through perfusion analysis, as a sensitive tool for evaluating microvascular flow, [18, 19].

CEUS may further enhance the assessment by detailing perfusion patterns in DTD, aiding in the precise diagnosis of thyrotoxicosis variants. CEUS is a versatile imaging modality with applications across various organ systems [20]. In addition to its established use in hepatic imaging—where it differentiates benign from malignant lesions through real-time vascular pattern analysis—CEUS [21] is applied in renal, pancreatic, breast and even cardiac imaging [22]. It enables detailed assessment of microvascular blood flow, aiding in characterizing lesions and inflammatory changes in these organs. In the thyroid, CEUS has shown potential in research settings for evaluating nodules and exploring vascular characteristics associated with malignancy [23–25]. However, despite its broad application, CEUS has not yet been used in diffuse thyroid diseases, where it could potentially offer valuable insights by visualizing diffuse tissue perfusion, which differs in conditions characterized by either hyperfunction or tissue destruction.

Identifying the underlying etiology of thyroid hormone excess is essential for selecting the most appropriate therapeutic approach [1]. The aim of this study is to evaluate the utility of CEUS in distinguishing between thyrotoxicosis caused by thyroid tissue destruction and hyperfunction. By assessing qualitative and quantitative CEUS parameters, we aim to establish specific vascular and perfusion characteristics that could aid in the differential diagnosis of these two pathophysiologically distinct forms of thyrotoxicosis.

## Material & methods

### Patients

A prospective, single-center study was conducted from January to September 2024. Sixty-two patients with newly diagnosed clinical thyrotoxicosis, prior to any treatment initiation, were enrolled and evaluated in the Ultrasound Department of a specialized endocrinology center in Timișoara, Romania. All patients presented with a clinical history indicative of thyrotoxicosis, supported by biochemical findings consistent with a hyperthyroid state, including low or suppressed thyroid-stimulating hormone (TSH) levels and elevated free T4 and/or T3. Patients were categorized into two groups based on the etiology of thyrotoxicosis: hyperfunctioning forms (Graves’ disease) and destructive forms (subacute thyroiditis, iatrogenic thyrotoxicosis, or Hashitoxicosis). The categorization was based on clinical findings, laboratory tests (detailed below), and technetium thyroid scintigraphy, which was performed in all patients to assess thyroid uptake. Scintigraphy served as a key reference standard for classification. Patients with thyrotoxicosis due to toxic adenoma, toxic multinodular goiter, or exogenous administration of levothyroxine were excluded from the study. Each participant provided written informed consent before joining the study. The research complied with the standards outlined in the Declaration of Helsinki, revised in 2000 in Edinburgh, and received approval from our institution’s Local Ethics Committee (approval nr. 61/11.12.2023).

### Contrast-enhanced ultrasound evaluation

The B-mode ultrasound examination was conducted to exclude nodular lesions and calculate thyroid volume. Also, diffuse hypoechogenicity of the parenchyma was noted, defined as thyroid parenchyma displaying decreased or similar echogenicity relative to the adjacent strap muscles on B-mode ultrasound. A longitudinal view of the thyroid lobe was obtained, followed by a CEUS examination using a Mindray Resona R9 ultrasound device equipped with an L15-3WU linear probe. In each patient, 1.4 mL of the contrast agent (Sonovue, Bracco; Milan, Italy) was injected into a peripheral vein, followed by 10 mL of 0.9% sodium chloride solution via the same intravenous line. A three-minute CEUS recording of the selected focal area was captured and stored in the device’s memory for quantitative analysis post-examination.

During the live CEUS evaluation, the examiner recorded key *qualitative parameters*, including the time of initial contrast arrival in the thyroid tissue (**wash-in start - WIS**), **time to peak intensity (TPI**), and **wash-out status 30** **s (Wo30)** post-injection. The CEUS examination and qualitative assessment of contrast-enhancement patterns were conducted by a single investigator with over five years of experience in thyroid ultrasound, with retrospective evaluation by the same investigator.

For *quantitative analysis*, perfusion parameters were assessed across three main categories [26]:

1. **Time Parameters:** These capture various time-related aspects of contrast agent dynamics:
  1. **Arrival Time (AT):** The time at which the Time-Intensity Curve (TIC) reaches a predefined level.
  2. **Rise Time or Wash-in Time (RT):** Calculated as RT = TTP - AT, indicating the interval from contrast arrival to peak intensity.
  3. **Time to Peak (TTP):** The point at which TIC reaches its peak intensity within the global Region of Interest (ROI).
  4. **Fall Time or Wash-out Time (FT):** Defined as FT = To - TTP, representing the time from peak intensity to clearance (To as the end of recording).
  5. **Mean Transit Time (mTT):** The period from contrast entry to clearance, calculated as mTT = RT + FT.
2. **Amplitude Parameter (PKI):** This measures the peak intensity of the TIC within the global ROI, offering insight into perfusion levels by showing the highest contrast enhancement.
3. **Amplitude-Time Combination Parameters:** These integrate intensity and timing aspects:
  1. **Area Under Curve (AUC):** Represents the area under TIC over the mTT period.
  2. **Wash-in AUC (WiAUC):** Area under TIC during RT, reflecting initial enhancement.
  3. **Wash-out AUC (WoAUC):** Area under TIC during FT, reflecting clearance dynamics.
  4. **Ascending Slope (AS):** Slope of enhancement from start to peak, calculated as (Imax – IAT)/RT.
  5. **Descending Slope (DS):** Slope of clearance from peak to end, calculated as (Imax - IAT)/FT.
  6. **Slope Ratio (SR):** The ratio of DS to AS, indicating relative enhancement and clearance rates.
  7. **Mean Time-Intensity Curve (mTIC):** Average intensity across the observation period, providing an overall contrast measure.

### Thyroid scintigraphy and laboratory evaluation

Technetium thyroid scintigraphy was performed to assess thyroid uptake and support the diagnosis of destructive thyrotoxicosis. Laboratory evaluation followed and was specific in each case. In all cases, TSH, free T4 (fT4), and free T3 (fT3) levels were measured using the Chemiluminescent Microparticle Immunoassay (CMIA) method. Values for fT3 and fT4 were reported relative to the upper limit of reference ranges to ensure consistent interpretation across cases. Subacute thyroiditis, suspected based on clinical findings such as anterior neck pain and tenderness, was further supported by elevated inflammatory markers—C-reactive protein (CRP) and/or erythrocyte sedimentation rate (ESR)—and confirmed by technetium scintigraphy, which showed low or absent thyroid uptake. The iatrogenic group included patients treated with amiodarone or immunotherapy, with other causes of thyrotoxicosis excluded. Hashitoxicosis was identified in hyperthyroid patients who had negative TRAb but positive anti-thyroid antibodies, specifically anti-thyroid peroxidase (ATPO) and/or anti-thyroglobulin (ATG) antibodies. ATPO levels were assessed by CMIA (normal range <34 IU/mL), while ATG levels were evaluated using electrochemiluminescence immunoassay (ECLIA; normal range <115 IU/mL). The second group comprised patients with Graves’ disease, confirmed by elevated TRAb levels, with a clinical decision limit of <1.75 IU/L (measured by ECLIA). TRAb levels were quantified using electrochemiluminescence immunoassay to confirm the etiology of Graves’ disease in these patients.

### Statistical analysis

Statistical analysis was conducted using MedCalc v12.5 software from MedCalc Software, Belgium. Descriptive statistics summarized demographic, clinical, and ultrasound data. The normality of numerical variables was assessed with the D’Agostino-Pearson test. Variables with normal distribution were expressed as mean and standard deviation, while non-normally distributed variables were represented by their median and interquartile range (IQR) from the 25th to 75th percentiles. Qualitative data were described using counts and percentages. The Mann-Whitney U-test was used for non-parametric variables, while parametric variables were analyzed with the t-test and ANOVA. Spearman’s correlation was employed to assess relationships between non-parametric variables; for associations between two dichotomous variables, the phi coefficient was used. To evaluate the diagnostic accuracy of quantitative CEUS parameters, receiver operating characteristic (ROC) curves were generated, and optimal threshold values were determined to distinguish between different pathologies. A *p*-value of <0.05 was considered statistically significant.

## Results

The study included a total of 42 cases, divided into two groups based on the underlying cause of thyrotoxicosis. Group 1, comprising hyperfunction cases, included 20 patients with confirmed Graves’ disease. Group 2 included 22 patients with destructive forms of thyrotoxicosis: 14 with subacute thyroiditis (SAT), 2 with Hashitoxicosis, 4 with postpartum thyroiditis, and 2 iatrogenic cases (1 due to amiodarone therapy and 1 due to immune therapy). Table 1 presents the thyroid function and ultrasound assessment in the two groups: patients with hyperfunction versus destructive forms of thyrotoxicosis.

**Table 1:** Thyroid function and ultrasound assessment in the two groups

	Hyperfunction	Destruction	*p*
Number of patients	20	22	–
Gender (F/M)	16/4	14/8	0.406
TSH	0.008 (0.005–0.08)	0.06 (0.02–0.1)	0.065
fT3	2.95 (1.65–4.6)	1.6 (1.2–3)	0.034
fT4	2.5 (2.05–3)	2.2 (2–3)	0.485
fT3/fT4	1.165 (0.826–1.4)	0.758 (0.480–0.917)	0.813
TV	24.9 (20–35.2)	14.6 (9–17.9)	<0.001
hypoechogenicity	19 (95%)	16 (72%)	0.128

*F* females, *M* males, *TV* thyroid volume

Table 2 displays the qualitative parameters obtained from CEUS assessments in the two groups, finding statistically significant differences between the two groups for all the four evaluated parameters.

**Table 2:** CEUS qualitative parameters

	Hyperfunction	Destruction	*p*
WIS	6 (5.5–7)	7.5 (7–8)	<0.001
TPI	13 (12–14)	15 (12–16)	0.001
Wo30	0 (0%)	21 (95.45%)	<0.001
IH	4 (20%)	19 (86.36%)	<0.001

*WIS* wash-in start, *TPI* time to peak intensity, *Wo30* wash-out status: more than 50% washout at 30 s, *IH* inhomogeneous enhancement

The most powerful correlation was observed with a Spearman correlation coefficient of 0.953 (*p* < 0.001) between the parameter “washout at 30 s” and the destructive type of thyroid disease. This strong positive correlation suggests that the washout at 30 s is a highly reliable indicator for distinguishing destructive thyroid conditions from hyperfunctional ones. the other qualitative parameters except for time to peak intensity of enhancement showed moderate correlations to the type of disease (0.637 for WIS and 0.666 for IH, *p* < 0.0001).

Table 3 presents the quantitative parameters derived from Contrast-Enhanced Ultrasound (CEUS) analysis in the two groups. The data highlight key differences in CEUS parameters between hyperfunction and destructive thyroid disease. Hyperfunction cases show significantly higher values for PKI, AUC, WiAUC, WoAUC, AS, DS, mTIC compared to the destruction group (all *p* < 0.001). Additionally, RT and TTP are notably prolonged in the destruction group (*p* = 0.001 and *p* < 0.001), while FT is slightly shorter (*p* = 0.008). No significant differences are observed in AT, mTT, or SR. These findings underscore the distinct enhancement patterns in hyperfunction versus destructive thyroid conditions, with CEUS providing clear diagnostic differentiation.

**Table 3:** CEUS quantitative parameters

	Hyperfunction	Destruction	*p*
AT	5 (3.80–5.65)	5.86 (4.89–6.12)	0.075
RT	7.31 (6.40–7.96)	9.47 (7.57–12.57)	0.001
TTP	12.35 (11.34–13.45)	14.92 (12.34–17.23)	<0.001
FT	167.68 (166.55–168.66)	164.44 (161.44–166.44)	0.008
mTT	175.22 (174.17–176.33)	174.09 (173.79–175.02)	0.093
PKI	40.67 (37.90–44.12)	25.44 (24.49–27.24)	<0.001
AUC	4445 (3642–5067)	1357 (1221–1767)	<0.001
WiAUC	226.5 (210–275)	151.33 (107–198.22)	<0.001
WoAUC	4109.5 (3442–4886)	1267.27 (1023–1651.91)	<0.001
AS	4.73 (4.56–5.04)	3.98 (2.78–4.23)	<0.001
DS	0.17 (0.15–0.18)	0.14 (0.13–0.15)	<0.001
SR	0.03 (0.03–0.04)	0.03 (0.03–0.05)	0.064
mTIC	23.67 (21.49–27.48)	8.35 (8.08–9.82)	<0.001

*AT* arrival time, *RT* rise time or wash-in time, *TTP* time to peak, *FT* fall time or wash-out time, *mTT* mean transit time, *PKI* amplitude parameter, *AUC* area under curve, *WiAUC* wash-in AUC, *WoAUC* wash-out AUC, *AS* ascending slope, *DS* descending slope, *SR* slope ratio, *mTIC* mean time-intensity curve

Strong positive correlations were observed between the type of disease (coded as 1 for hyperfunctioning and 0 for destructive) and the perfusion parameters PKI (ρ = 0.867), AUC (ρ = 0.866), WoAUC (ρ = 0.866), mTIC (ρ = 0.867), and WiAUC (ρ = 0.706), indicating higher values of these parameters in hyperfunctioning cases. Moderate negative correlations were found for RT (ρ = −0.545) and TTP (ρ = −0.555) (*p* < 0.001 for all), suggesting these parameters were lower in hyperfunctioning compared to destructive forms.

**Fig. 1:**
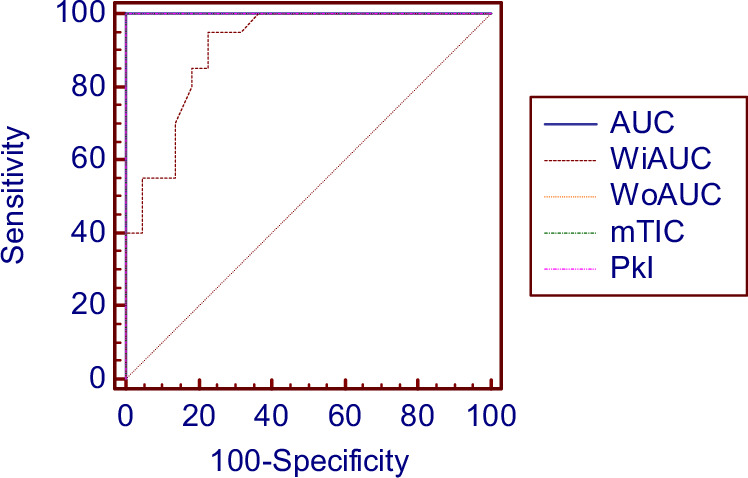
Receiver Operating Characteristic (ROC) Curves for Quantitative CEUS Parameters in Differentiating Hyperfunctional and Destructive Thyrotoxicosis. AUC Area under Curve, WiAUC Wash-in AUC, WoAUC Wash-out AUC, mTIC Mean Time-Intensity Curve, PKI Amplitude parameter

Considering these findings, we further evaluated the optimal cut-off values for distinguishing between hyperfunctional and destructive forms of thyrotoxicosis (Table 4). For a cut-off value of AUC > 1991, sensitivity and specificity reached 100% for detecting hyperfunctional forms (Fig. 1). For WiAUC > 198.22, sensitivity was 95% and specificity 77.3%. Additionally, cut-off values for WoAUC > 1876 and mTIC > 10, PKI > 28 dB, all three achieved sensitivity and specificity of 100%, indicating strong diagnostic accuracy in differentiating the two forms.

**Table 4:** Cut-off values and ROC statistics for quantitative CEUS parameters in differentiating hyperfunctional and destructive thyrotoxicosis

	Optimal cut-off value	AUC	Sensitivity	95% CI	Specificity	95% CI
AUC	>1991	1	100%	83.2–100	100%	84.6–100
mTIC	>10	1	100%	83.2–100	100%	84.6–100
PkI	>28	1	100%	83.2–100	100%	84.6–100
AT	<5.7	0.653	80%	56.3–94.3	54.55%	32.2–75.6
RT	<8.7	0.768	90%	68.3–98.8	59%	36.4–79.3
TTP	13.6	0.818	100%	83.2–100	59%	36.4–79.3
WiAUC	>198	0.907	95%	75.1–99.9	68.18%	45.1–86.1
WoAUC	>1876	1	100%	83.2–100	100%	84.6–100
AS	>4.36	0.869	80%	56.4–94.3	86.36%	65.1–97.1
DS	>0.14	0.877	100%	83.2–100	63.64%	40.7–82.8
SR	<0.04	0.619	85%	62.1–96.8	40.91%	20.7–63.6
FT	>165.6	0.868	100%	83.2–100	68.18%	45.1–86.1
mTT	>175.1	0.698	50%	27.2–72.8	86.36%	65.1–97.1

*AUC* area under curve, *CI* confidence interval, *mTIC* mean time-intensity curve, *PKI* amplitude parameter, *AT* arrival time, *RT* rise time or wash-in time, *TTP* time to peak, *FT* fall time or wash-out time, *mTT* mean transit time, *AUC* area under curve, *WiAUC* wash-in AUC, *WoAUC* wash-out AUC, *AS* ascending slope, *DS* descending slope, *SR* slope ratio

**Fig. 2:**
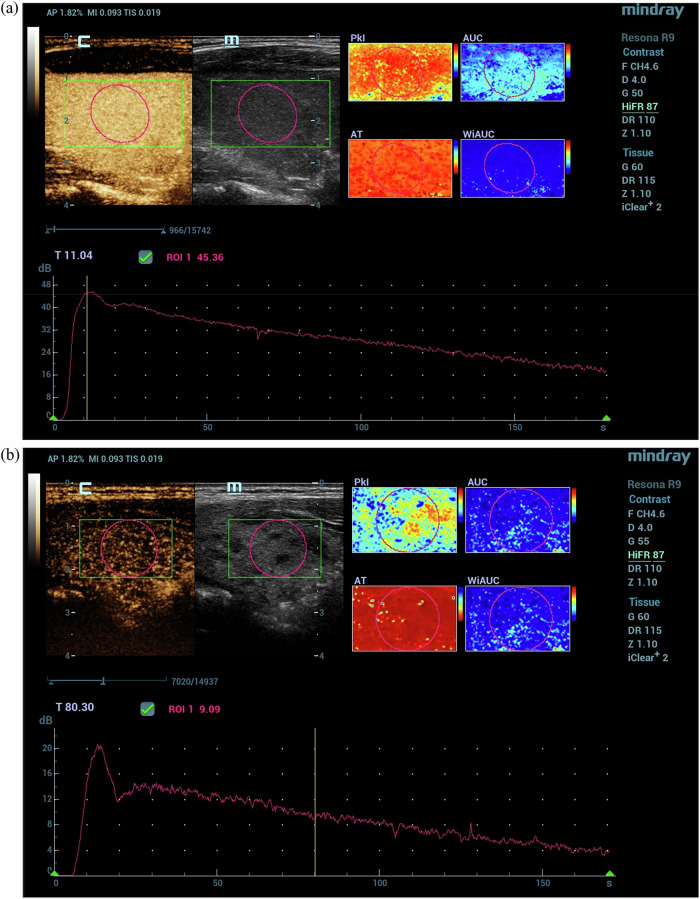
Quantitative CEUS – Time intensity curve analysis with left upper corner of the image displaying CEUS map) left and B-mode image (right), the right upper corner displaying quantitative paramers map for the selected region of interest and the inferior half of the image displaying the time intensity curve for CEUS in the selected area: (**a**) in a case with hyperfunction caused by Graves’ disease and (**b**) in a case with destruction caused by subacute thyroiditis

Figure 2 presents the CEUS images and corresponding time-intensity curves for a patient with Graves’ disease (a) and a patient with subacute thyroiditis (b). The differences in enhancement patterns and vascularity are visually apparent, with the hyperfunctional Graves’ disease showing distinct contrast dynamics compared to the destructive subacute thyroiditis.

## Discussion

Other Doppler-based ultrasound parameters have been proposed to aid in differentiating hyperfunctional and destructive forms of thyrotoxicosis. One common qualitative approach, the visual Doppler scale, evaluates thyroid vascularity but lacks quantitative precision [19]. Doppler ultrasound demonstrated a sensitivity of 88.9%, specificity of 87.5% and diagnostic accuracy of 88.5% for differentiating Graves’ disease from Hashimoto’s thyroiditis when compared to 99mTc scintigraphy [8]. Similarly, Color Doppler scale ultrasound achieved a sensitivity of 96% and specificity of 95% in differentiating types of thyrotoxicosis, also using 99mTc scintigraphy as a reference [27].

Quantitative Doppler measures, such as peak systolic velocity (PSV), have also been explored. In the literature, PSV values over 40–50 cm/sec [8, 18, 28, 29] have been identified as effective thresholds, with diagnostic accuracy reaching 88%, comparable to thyroid scintigraphy. Notably, PSV values for Graves’ disease are consistently higher than those for thyroiditis, providing a useful marker for differential diagnosis.

Beyond Doppler ultrasound, elastography is another significant ultrasound-based tool for thyroid evaluation [30–34]. Elastography has shown marked differences in elasticity values between subacute thyroiditis (SAT) and other types of thyroiditis [10]. For example, 2D shear wave elastography (SWE) reveals mean elasticity values over 150 kPa in SAT, while autoimmune diseases like Graves’ disease and Hashimoto’s thyroiditis exhibit much lower stiffness values, around 25 kPa [12]. Furthermore, strain elastography can also aid in differentiation; for instance, strain ratios (SR) for patients with Hashimoto’s and Graves’ disease versus SAT have been shown to be effective, with a cut-off SR of 14.79 providing 80% sensitivity and 85% specificity (AUC 0.869, 95% CI) [35]. These findings highlight the added diagnostic value of elastography in distinguishing SAT from other forms of diffuse thyroid disease.

In this study, we demonstrated the effectiveness of CEUS in distinguishing between hyperfunctional and destructive forms of thyrotoxicosis. The observed significant differences in CEUS parameters, such as AUC, WiAUC, WoAUC, and mTIC, provide valuable insight into the distinct vascular characteristics of these thyroid conditions. Hyperfunctional thyroid disease, as seen in Graves’ disease, displayed higher peak intensity, faster wash-in, and prolonged enhancement, which correlates with increased blood flow typical of hyperfunction. Conversely, destructive forms, such as subacute thyroiditis, exhibited lower enhancement and faster wash-out, reflecting reduced or absent vascular perfusion due to tissue destruction. The strong correlations observed for AUC, WoAUC, and mTIC with the type of thyrotoxicosis further reinforce the diagnostic value of these parameters. The cut-off values identified, particularly with PKI > 28 dB, AUC > 1991 and mTIC > 10 achieving 100% sensitivity and specificity for detecting hyperfunctional forms, underscore the potential of CEUS as a precise, non-invasive diagnostic tool in differentiating these pathophysiologically distinct forms of thyroid disease.

These findings are clinically relevant, as accurate differentiation between hyperfunction and destruction-based thyrotoxicosis can guide appropriate treatment strategies, reducing the risk of unnecessary interventions. For example, patients with destructive thyroiditis may avoid antithyroid medications, which are not indicated in the absence of hyperfunction. Currently, no comparable studies are available in the literature, making this investigation a novel contribution to the field. Traditionally, scintigraphy has been the standard method for distinguishing between hyperfunctional and destructive forms of thyrotoxicosis [36]. The aim of the study is to explore CEUS as an alternative diagnostic tool that, in some cases, may be more easily applicable than scintigraphy in the complex diagnostic process of thyrotoxicosis—particularly in distinguishing between destructive and hyperfunctional forms—while still recognizing the pivotal role of scintigraphy as the reference standard in the diagnostic workup of hyperthyroidism.

This study has several limitations. First, CEUS availability may still be limited in some clinical settings due to cost and the need for specialized equipment or contrast agents. Additionally, in certain European countries, thyroid imaging is not an approved indication for contrast-enhanced ultrasound, which may restrict its immediate clinical application. However, CEUS is generally becoming more accessible than scintigraphy, which remains costly, less available, and often declined by patients due to radiation concerns. In centers where CEUS is available, it offers a rapid, office-based diagnostic alternative. Second, the diagnostic accuracy of both CEUS and scintigraphy can be influenced by the timing of the examination, particularly in dynamic conditions like subacute thyroiditis, where the disease evolves through destructive and regrowth phases. Another limitation of this study is that all CEUS examinations were performed and interpreted by a single experienced operator. While this ensured consistency in technique and interpretation, it may introduce operator-related bias. Lastly, the heterogeneity of the destructive group—encompassing multiple thyrotoxicosis mechanisms—may influence the perfusion profiles observed and should be addressed in future studies. Due to small subgroup sizes, we could not perform a separate perfusion analysis for each etiology (e.g., SAT vs. Hashitoxicosis). It remains possible that perfusion patterns vary among these entities, and future studies with larger cohorts are needed to explore these differences. Although several CEUS parameters demonstrated high diagnostic performance in this study, including sensitivities and specificities approaching 100%, these values should be interpreted with caution given the small sample size and absence of external validation.

This study represents an exploratory, single-center investigation aimed at assessing the potential application of CEUS in differentiating hyperfunctional and destructive forms of thyrotoxicosis. Given the limited sample size and the heterogeneity of the destructive group, the findings should be interpreted as preliminary. Nonetheless, the results provide a promising foundation for future research. Validation in larger, multicenter cohorts is essential to confirm diagnostic cut-off values and further establish CEUS as a standardized diagnostic tool in diffuse thyroid disease.

## Conclusion

In conclusion, both qualitative and quantitative CEUS parameters demonstrate strong potential in distinguishing between hyperfunctional and destructive forms of thyrotoxicosis. The high sensitivity and specificity of parameters such as AUC, WoAUC, and mTIC reinforce CEUS as a valuable, non-invasive diagnostic tool that supports rapid diagnosis and accurate selection of treatment strategies for DTD associated with hyperfunction.

## Data Availability

No datasets were generated or analysed during the current study.
